# Identification of arboviruses in mosquito populations in KwaZulu-Natal, South Africa and the first record of *Wyeomyia mitchellii* in the Old World

**DOI:** 10.1371/journal.pntd.0013093

**Published:** 2025-08-12

**Authors:** Nosipho Z. Masoto, Phillip A. Bester, Louwrens P. Snyman, Natasha Govender, Danelle van Jaarsveldt, Felicity J. Burt

**Affiliations:** 1 Division of Virology, University of the Free State, Bloemfontein, South Africa; 2 Division of Virology, National Health Laboratory Service, Universitas, Bloemfontein, South Africa; 3 Invertebrate Zoology Department, Royal Alberta Museum, Edmonton, Alberta, Canada; 4 University of Saskatchewan, Saskatoon, Saskatchewan, Canada; 5 Entomology Department, Durban Natural Science Museum, eThekwini, South Africa; Medizinische Universitat Wien, AUSTRIA

## Abstract

Mosquito-borne viruses have the potential to spread and cause outbreaks with significant public and veterinary health consequences. Although historically a significant number of arboviruses were identified in South Africa with potential to cause sporadic outbreaks, there is limited information on the current situation in some regions of the country. Hence a study was initiated to investigate which arboviruses are currently circulating within mosquito populations in a major metropolitan area, eThekwini, KwaZulu-Natal Province. Mosquitoes were collected from seven sites throughout the metropole and a subset were screened for arboviruses from the families *Togaviridae*, *Phenuiviridae* and *Peribunyaviridae.* The subset of 1831 mosquitoes were collected between October 2020 and July 2021, identified morphologically, and pooled according to species, collection site and collection date. RNA was extracted from a total of 261 mosquito pools and screened using in-house nested and hemi-nested reverse transcriptase polymerase chain reaction (RT-PCR). Primers targeting conserved genes for each viral genus were used in a nested or hemi-nested two-step RT-PCR. Amplicons were sequenced to determine the virus species. Arboviral RNA was detected from 15/261 mosquito pools. The amplicons were subsequently sequenced using the Oxford Nanopore MinION. The positive samples included a Sindbis virus (SINV) isolate, three isolates of Witwatersrand virus (WITV), and 11 isolates of Bunyamwera virus (BUNV). Phylogenetic analysis of partial sequence data suggested that none were newly introduced but closely related isolates previously detected in the country. SINV is known to cause outbreaks of human disease after heavy rainfall, favoring an increase in mosquito populations. Bunyamwera virus has been associated with human febrile disease, but severe disease and regular outbreaks have not been reported previously and requires further investigation. The medical significance of WITV is currently unknown. *Wyeomyia mitchellii*, a New World species, is for the first time confirmed as an introduced species in South Africa and highlights the importance of vector surveillance. Identification of circulating viruses and raising the awareness of the presence of these viruses is important for early detection and determining the public health significance.

## Author summary

Historically, several arboviruses have been identified in South Africa, however, some of this surveillance was performed more than 40 years ago. In this study mosquito populations from a major metropolitan area, eThekwini, KwaZulu-Natal Province were screened for arboviruses from the families *Togaviridae*, *Phenuiviridae* and *Peribunyaviridae*. Wild caught mosquitoes were collected from seven sites, between October 202 and July 2021, throughout the metropole and a subset (1831) were screened using in-house nucleic acid amplification assays and primers targeting conserved genes for each viral genus. Arboviral RNA was detected from 15/261 mosquito pools and amplicons sequenced using the Oxford Nanopore MinION. The positive samples included a Sindbis virus isolate, three isolates of Witwatersrand virus, and 11 isolates of Bunyamwera virus. Phylogenetic analysis of partial sequence data suggested that none were newly introduced but closely related to isolates previously detected in the country. The results highlight the importance of ongoing surveillance to monitor arbovirus circulation. In addition, we report the presence of *Wyeomyia mitchellii*, a New World species, as an introduced species in South Africa. Identification of circulating viruses and raising the awareness of the presence of these viruses is important for early detection and determining the public health significance.

## Introduction

Several zoonotic mosquito-borne viruses of medical and veterinary importance are endemic to South Africa (SA), including viruses from the families *Flaviviridae (*genus *Orthoflavivirus) Togaviridae* (genus *Alphavirus), Phenuiviridae* (genus *Phlebovirus*) and *Peribunyaviridae* (genus *Orthobunyavirus*) [1]. West Nile virus (WNV) and Sindbis virus (SINV) are most frequently detected as a cause of human disease with outbreaks usually following periods of heavy rainfall that favors mosquito breeding [2]. Wesselsbron virus (WSLV), although less frequently identified, causes disease in domestic livestock and humans, often co-circulating with Rift Vally fever virus (RVFV). However historically there are other arboviruses that have been identified in the country, mostly from vector surveillance, but the current circulation and medical significance are unknown. Although some of these viruses may be less significant on the African continent and considered to be circulating within immunologically adapted populations, there is potential for spread and emergence in new geographic regions with public health implications. Zika virus and chikungunya virus (CHIKV) are examples of viruses that have spread from the Old World and become established in New World countries causing significant disease [3,4].

Historical reports of evidence of less well-known arboviruses in the country have largely been based on virus isolation from mosquitoes and detection of antibodies in human populations during serosurveys, with absence of identification of acute cases and associated disease. Less common alphaviruses known to occur in SA include Middelburg virus (MIDV) and Ndumu virus (NDUV) [2]. MIDV was initially identified in 1957 as a cause of disease among sheep in the Middelburg region of SA and has more recently been associated with neurologic disease with fatalities in horses [5]. Although serological surveys suggest human exposure, the medical significance is unknown [6]. Similarly, NDUV was isolated in the 1960s from mosquitoes in KwaZulu-Natal (KZN) and antibodies were detected in human serosurveillance studies, but acute cases have not been described [1]. CHIKV is not known to be endemic to SA, although human infections were recorded in the Eastern Transvaal (currently known as Mpumalanga Province) in 1956, 1974, 1976, and 1977. Even though the virus has not been associated with locally acquired disease since then, there is potential for reemergence in the region [7].

Viruses from the order *Bunyavirales* such as Bunyamwera virus (BUNV), Shuni virus (SHUV), Witwatersrand virus (WITV), Germiston virus (GERV) and RVFV have all been detected in the country [8–12]. This order includes some viruses that are known to be pathogenic to humans causing diseases ranging from self-limited fever to severe haemorrhagic fever. SHUV has been associated with neurological disease in humans and horses and RVFV causes spontaneous abortion in animals, and human disease in ~1% of infected people. BUNV, in turn, is generally associated with mild disease characterised by joint pain and rash in humans, and severe disease has not yet been described. The potential for disease warrants further investigation into the prevalence of members of the *Orthobunyavirus* genus in SA.

Surveillance plays an important role in early detection and increased awareness of pathogens with epidemic potential. Hence the importance of establishing surveillance programs to further understand the dynamics of arbovirus circulation. In this study, a subset of mosquitoes that were collected throughout a major metropolitan area, eThekwini, KZN province in SA were retrospectively screened for a range of arboviruses to target less frequently detected viruses that were identified historically.

## Methods

### Ethics statement

This study was approved by the Environmental and Biosafety Research Ethics Committee of University of the Free State, Ethical clearance number: UFS-ESD2021/0205/21.

A section 20 permit was granted from Department of Agriculture Land Reform and Rural Development (DALRRD) to collect and screen mosquitoes for arboviral infections.

### Mosquito sampling and identification

**Fig 1:**
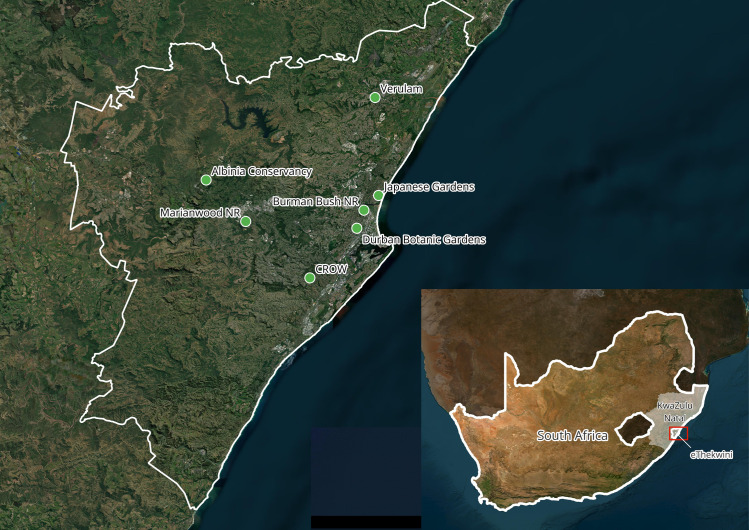
A map of the mosquito collection sites in eThekwini, KZN. The map was constructed in QGIS 3.28.0 using an ESRI satellite basemap (ESRI 2025). NR: Nature Reserve; CROW: Centre for Rehabilitation of Wildlife. References: ESRI. “ESRI Satellite” [basemap]. Scale Not Given. World Imagery. Original date not provided. https://server.arcgisonline.com/ArcGIS/rest/services/World_Imagery/MapServer/tile/{z}/{y}/{x} (Accessed 23 January 2025).

Mosquitoes were collected from seven sites in and around Durban (eThekwini municipality) as part of a mosquito biodiversity project. The coastal metropole is located on the east coast of South Africa in KwaZulu-Natal. The Municipality spans an area of approximately 2300km^2^ and is home to some 3,5 million people, the third most populous in the country. The collection sites that were selected are Albinia Conservancy (AC), Burman Bush Nature Reserve (BBNR), Durban Botanic Gardens (DBG), Japanese Gardens (JG), Mariannwood Nature Reserve MWNR), Centre for Rehabilitation of Wildlife (CROW) and an urban site in Verulam (Ver) (Fig 1). Mosquitoes were collected at least once a month at each site between October 2020 and July 2021. KwaZulu Natal province has a humid subtropical climate with summer rainfall and an average temperature of 24°C (75°F) in summer months (October to April) and 17°C (63°F) in winter months. Mosquito populations can remain active throughout the year, peaking in summer, due to the mild conditions. Collections were performed using Encephalitis-Virus-Surveillance (EVS) traps and Biogents Sentinel 2 (BG) traps. All traps were baited with ±1 kg of dry ice before being deployed late in the afternoon and retrieved each morning. Captured mosquitoes were kept frozen until processing.

The mosquitoes were identified morphologically using the keys of Jupp [13] and Gillies & Coetzee [14]. Identified specimens were pooled to a maximum of 60 specimens according to species, collection site and date of collection and stored at -80°C until transported to the Pathogen Research Laboratory at the Division of Virology, University of the Free State. A total of 261 mosquito pools, a subset of the total catch, including mosquitoes from the genera *Aedes, Culex, Eretmapodites, Mansonia, Lutzia* and *Wyeomyia*, were screened for arboviral RNA using molecular techniques.

### Viral RNA extraction and nested RT-PCR

Viral RNA was extracted from mosquito pools in the biosafety level 2 laboratory at the Pathogen Research Laboratory, Division of Virology, University of the Free State. Briefly, each mosquito pool containing 30 or less mosquitoes was homogenized in a 500µl aliquot of Dulbecco’s Modified Eagle Medium (DMEM) supplemented with 10% foetal bovine serum (FBS), 0.1% mg/ml penicillin/streptomycin and 2mM L-glutamine. Larger mosquito pools (up to 60 per pool) were homogenized using 700µl of media. A 100µl aliquot of each homogenate was stored at -80°C for future virus isolation attempts and RNA extracted from the remaining aliquot using 600µl TRIzol Reagent (ThermoScientific, USA) and RNeasy kit (Qiagen, Germany) following manufacturer’s instructions.

cDNA was prepared using Protoscript II first-strand cDNA synthesis kit (New England Biolabs (NEB), United States of America (USA)). A 20µl reaction mix was prepared which included 10µl Protoscript II reaction mixture, 2µl Protoscript enzyme mixture, 0.5µl of each forward (F1) primer listed in Table 1, a 3µl aliquot of RNA extracted from mosquitoes and 2.5µl of nuclease-free water. Primers were designed using multiple alignments of sequence data for selected alphaviruses, and orthobunyaviruses publicly available data (S1 Table).

**Table 1:** Primers used for cDNA synthesis and first round RT-PCR.

Virus or genus/ targeted partial gene	Primer name	5’ – 3’ Primer sequence (Primer position on reference strain)	Annealing temperature	Amplicon size (bp)
*Alphavirus/* NsP4	Alphavirus nsP4 F1^a^	AARTTYGGSGCSATGATGAA (6984-7004)	54°C	570
	Alphavirus nsP4 R1^a^	CWATTTAGGACCRCCGTASA (7553-7534)		
Bunyamwera virus (BUNV) & Germiston virus (GERV)/ S segment	BUNV & GERV F1^b^	CRGSAGTWCTTTTGACCCAGAG (42-60)	58°C	407
	BUNV & GERV R1^b^	ACCKGGCAAGGWATCCACTGA (310-284)		
Shuni virus (SHUV) & Witwatersrand virus (WITV)/ S segment	SHUV & WITV F1^b^	TACRTTTAACCCKGAGGTC (74-92)	54°C	266
	SHUV & WITV R1^b^	CCARGAACATTTCWGYACCTGG (483-462)		
Rift Valley Fever Virus (RVFV)/ M segment	RVFV F1 (009)	CCAAATGACTACCAGTCAGC (771-790)	53°C	330
	RVFV R (007)^c^	GACAAATGAGTCTGGTAGCA (1139-1120)		

^a^ [15].

^b^ [16].

^c^ [17].

A conventional nested and hemi-nested PCR was performed using GoTaq DNA polymerase kit (Promega, USA) according to manufacturer’s instructions using primers targeting genes of interest (Tables 1 and 2). The cycling conditions were as follows: initial denaturation at 95°C for two minutes, followed by 30 cycles of denaturation at 95°C for one minute, annealing for one minute using temperatures indicated in Tables 1 and 2, and elongation at 72°C for one minute. This was followed be a final extension at 72°C for five minutes.

**Table 2:** Primers used for hemi-nested PCR.

Virus or genus/ targeted gene	Primer name	5’ – 3’ Primer sequence (Primer position on reference strain)	Annealing temperature	Amplicon size (bp)
*Alphavirus*/ NsP4	Alphavirus nsP4 F2^a^	GCSATGATGAARTCYGGHATG (6993-7014)	54°C	200
	Alphavirus nsP4 R2^a^	TTMACYTCCATGTTSAKCCA (7189-7173)		
Bunyamwera virus (BUNV) & Germiston virus (GERV)/ S segment	BUNV & GERV F2^b^	GACCAMATWCGMATCTTCTACA (109-130)	46°C	184
	BUNV & GERV R2^b^	ATCCACTGAKDCKGTGGA (191-172)		
Shuni virus (SHUV) & Witwatersrand virus (WITV)/ S segment	SHUV & WITV F2^b^	CGTTAGAKYCTTCTTCCTCMA (143-163)	53°C	186
	SHUV & WITV R2^b^	GCWAGATATCCKGAGAKRCG (328-314)		
Rift Valley Fever virus (RVFV)/ M segment	RVFV F2 (G)	AAAAAGTGTGATGGCCAACTC (1008-1028)	53°C	100
	RVFV R (007)^c^	GACAAATGAGTCTGGTAGCA (1139-1120)		

^a^ [15].

^b^ [16].

^c^ [17].

Nested RT-PCR products were separated using 1% agarose gel electrophoresis, or 2% agarose gel for amplicons <200 bp. DNA fragments were visualised using GelRed DNA stain and GelDoc system with Image Lab Software (Bio-Rad, USA).

### Positive controls for RT-PCR

RNA extracted from SINV infected cells was used as a positive control for the alphavirus RT-PCR. Transcribed RNA prepared from pGEM-T Easy vector plasmids containing partial synthetic S genes for SHUV, and BUNV was used for the orthobunyavirus RT-PCR. Briefly, RNA was transcribed from the SP6 promotor site on linearised plasmids using MEGAscript kit (ThermoScientific, USA) according to manufacturers’ instructions. Transcribed RNA was purified using SV Total RNA isolation kit (Promega, USA) according to manufacturers’ instructions.

### MinION library preparation and sequencing

The nucleotide sequence of positive amplicons was determined using MinION sequencing and the Oxford Nanopore Technologies (ONT) Ligation Sequencing kit (SQK-LSK109) version R9 (ONT, Oxford, United Kingdom (UK)) according to the nCoV-2019 sequencing protocol v3 (LoCost) [18]. The library preparation included three steps, template ends preparation, barcode ligation and adapter ligation. In short, the end preparation reaction was prepared for each of the 24 amplicons as follows: 3.3µl of amplicon, 1.2µl of Ultra II End preparation buffer (NEBNext Ultra II End Repair/dA-tailing Module, NEB, USA), 0.5µl of Ultra II End prep enzyme mix (NEBNext Ultra II End Repair/dA-tailing Module, NEB, USA) and 5µl of nuclease free water. The reactions were incubated at 21°C for 20 minutes, followed by 65°C for 15 minutes.

For the barcode ligation, the Native Barcoding kits EXP-NBD104 & EXP-NBD114 (ONT, Oxford, UK) were used. For each of the 24 end-prep reaction, a 1.5µl aliquot of the end-prep product was combined with 5µl of Blunt/TA Ligase Master Mix (NEB, USA), 1.5µl of native barcode and 2µl of nuclease free water. The samples were incubated at 21°C for 20 minutes, followed by 65°C for 10 minutes. A 10µl aliquot of each of the 24 barcoded samples were pooled into a single 1.5 ml centrifuge tube. A 96µl aliquot of SPRIselect Bead-based reagent (Beckman Coulter, USA) was added to the pooled reaction and mixed by pipetting. The bead mixture was incubated at room temperature for 10 minutes and pelleted by centrifugation, followed by magnetic separation of the beads. The beads were washed twice using 250µl of Short Fragment Buffer (SFB) (Part of SQK-LSK109 kit, ONT, Oxford, UK) and lastly with 200µl freshly prepared 80% ethanol. The pellet was resuspended using 32µl of nuclease-free water and incubated at 21°C for 10 minutes. The beads were pelleted on the magnetic rack and the clear supernatant transferred to a 1.5ml Eppendorf DNA LoBind tube.

For adapter ligation, a 30µl aliquot of the barcoded amplicon pool was added to 5µl of Adapter Mix II (AMII) (Part of SQK-LSK109 kit, ONT, Oxford, UK) and 35µl of Blunt/TA Ligase Master Mix (NEB, USA) and incubated at 21°C for 20 minutes, followed by 65°C for 10 minutes. A 140µl aliquot of SPRIselect Bead-based reagent (Beckman Coulter, USA) was added to the reaction and the reaction was incubated on a rotator mixer for 10 minutes at room temperature. The reaction was pelleted by centrifugation, followed by magnetic separation of the beads. The beads were washed twice using 250µl of SFB (Part of SQK-LSK109 kit, ONT, Oxford, UK). Finally, the pellet was resuspended in 15µl of Elution buffer (Part of SQK-LSK109 kit, ONT, Oxford, UK) and incubated at 21°C for 10 minutes. The beads were pelleted on the magnetic rack and the clear supernatant transferred to a 1.5ml Eppendorf DNA LoBind tube.

A 30µl aliquot of Flush tether (Part of Flow cell priming kit, EXP-FLP002, ONT, Oxford, UK) was added to a Flush buffer (Part of Flow cell priming kit, EXP-FLP002, ONT, Oxford, UK) tube and mixed by vortex. A MinION flow cell (R9.4.1) (FLO-MIN106D, ONT, Oxford, UK) was primed using an 800µl aliquot of the Flush buffer & tether mixture, followed by another 200µl aliquot to initiate a siphon at the SpotON port, which allows loading of the DNA library. To prepare the loading reaction, a 25.5µl aliquot of loading beads (Part of SQK-LSK109 kit, ONT, Oxford, UK) was completely resuspended in 37.5µl sequencing buffer (Part of SQK-LSK109 kit, ONT, Oxford, UK). A 12µl aliquot of DNA library was added to the mixture and the entire 75µl was loaded onto the primed MinION Flow cell.

### Data handling

The fast5 files were base-called using Guppy (v 6.1.7, ONT, Oxford, UK) on a server with 8 × NVIDIA V100 cards (eResearch & HPC unit of the University of the Free State) using the super accurate configuration file for revision 10.4.1. The base-called reads were mapped to reference sequences with accession numbers; M19420.1, H484290, NC_043673.1 and OK539682.1 for GERV, BUNV, WITV and SINV respectively. Minimap2 (2.26-r1175) was used to conduct the mapping of reads against the above-mentioned references. Samtools (1.18) and iVar (1.4.2) was used for primer removal and draft sequence calling followed by consensus correction and polishing using medaka (1.11.3) (ONT, Oxford, UK) to generate the final consensus sequences. The sequences were analysed using Basic Local Alignment Search Tool (BLAST) analysis (https://blast.ncbi.nlm.nih.gov/Blast.cgi).

### Analysis of similarity

Sequence analysis was performed for each positive amplicon detected in the mosquito pools using sequence data retrieved from GenBank. Briefly, nucleotide sequences for the partial genes of each virus detected by RT-PCR and representing as many lineages as possible and/or genotypes or geographically distinct isolates were retrieved from GenBank and were aligned with the partial gene sequences obtained in this study. Sequence alignment was performed using MAFFT version 7.490 with L-INS-I algorithm. The terminal gaps were removed from the aligned sequences using trimAL version 1.4 [19]. Clustal Omega was used to generate an identity matrix on the alignment [20]. The Seaborn Python library was used to generate a clustermap using the average (UPGMA) method as implemented by SciPy [21,22].

GenBank accession data are provided in S3 Table

### Molecular barcoding for mosquito species

One mosquito species was collected which has not previously been documented in SA and hence molecular barcoding was used to confirm the morphological identification.

Briefly, DNA was extracted from the homogenized mosquito pool using DNeasy blood and tissue kit according to the manufacturer’s instructions (QIAGEN, Valencia, CA, USA) and eluted in 50µl elution buffer (Buffer AE) and stored at -80°C until processing. A PCR was performed using extracted DNA as template. A partial gene fragment (709 bp) of CO1 was amplified using (LCO1490-5’GGTCAACAAATCATAAAGATATTGG3’) and (HCO-2198 5’TAAACTTCAGGGTGACCAAAAAATCA3’) primers [23]. PCR was performed using Phusion High-Fidelity (HF) DNA polymerase enzyme according to manufacturer’s instructions (NEB, USA). A 50µl PCR reaction was prepared using 31.5µl nuclease free water (NFW), 10µl 5x Phusion HF buffer, 1µl PCR nucleotide mix (10mM each), 1μl forward primer (20 pmol/μl), 1μl reverse primer (20 pmol/μl), 5μl template DNA and 0.5µl Phusion DNA polymerase (2 units/μl). The PCR reaction was cycled using Proflex PCR system (Applied Biosystems, New York, USA) and the following cycling conditions: initial denaturation at 98˚C for 30 seconds, 35 cycles of denaturation at 98˚C for 30 seconds, annealing at 52˚C for 45 seconds, elongation at 72˚C for 30 seconds, and a final elongation at 72˚C for five minutes before resting at 4°C. PCR products were visualised on a 1% agarose gel post-stained using GelRed Nucleic Acid Gel Stain 10000X solution (Biotium Inc, Hayward, USA) for 30 minutes on a shaker. The 1% agarose gel was visualized on an Image Lab Software (Bio-Rad, USA) using a Molecular Imager Gel Doc XR System (BioRad, USA). The nucleotide sequence of the amplicon was determined using Oxford Nanopore MinION sequencing (ONT, Oxford, UK).

## Results

### Confirmation of mosquito species *Wyeomyia mitchellii* (Theobald, 1905)

Four pools of mosquitoes collected in the Durban Botanical Gardens could not be identified using the keys to South African species. A BLAST search resulted in a 99% similarity with other publicly available *Wyeomyia mitchellii* CO1 sequences (S2 Table). The identification of a preserved specimen collected in the Durban Botanical Gardens as *Wyeomyia mitchellii* was confirmed following the keys of Darsie and Ward [24]. The specimen is deposited in the KwaZulu-Natal Museum with catalogue number NMSA-DIP 086656. The specimen could not be photographed for this study. This is the first record of the genus and species for the Old World.

### Detection of arboviral RNA in mosquito pools

**Fig 2:**
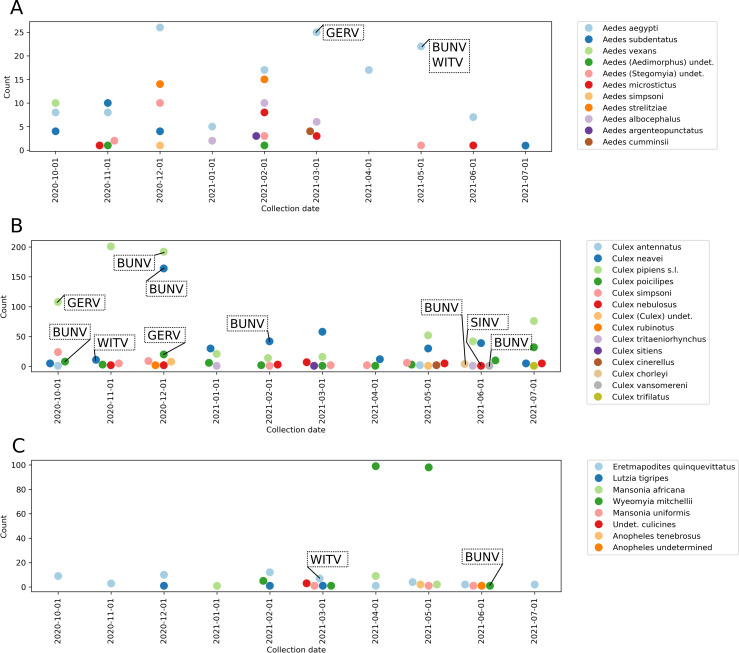
Mosquito collections and detection of positive pools. **A)** Dates on which *Aedes* spp. mosquitoes were collected, B) dates on which *Culex* spp. mosquitoes were collected, C) dates on which mosquito species from genera other than *Aedes* and *Culex* were collected. Pools in which positive amplicons were detected are indicated with black arrows and the name of the virus species.

A total of 1831 mosquitoes collected from all sites throughout the surveillance period were screened for selected arboviruses known to occur or with a potential to occur in SA (Table 3). This total resulted in 261 pools following morphological identification and pooling (Fig 2). The nested RT-PCR products were analysed using gel electrophoresis and amplicons were detected in 15 pools, including 1/15 positive for an alphavirus, and 14/15 positive for orthobunyaviruses (Table 4). Sindbis virus RNA was detected in one *Cx. nebulosus* specimen collected at BBNR. Three pools of mosquitoes, *Er. quinquevittatus, Cx. neavei* and *Ae. aegypti*, collected from different sites, AC, BBNR and Ver respectively, were each positive for WITV RNA. Germiston virus RNA was detected in *Culex* species collected from two different sites, BBNR and DBG and from *Ae. aegypti* at JG. The most frequently detected virus was BUNV, amplified from eight mosquito pools collected from four sites, JR, Ver, DBG and MNR. All positive amplicons were subsequently sequenced. The collection dates relative to the detection of positive reactors are shown in Fig 2. Briefly, although positive samples were detected from mosquito pools throughout the collection period, the frequency of detection was higher in collections performed from October to December in 2020.

**Table 3:** Mosquito samples collected throughout eThekwini Municipality that was screened for various arboviruses.

	Sites							
Species	**DBG**	**MWNR**	**AC**	**JG**	**Ver**	**BBNR**	**CROW**	**Total**
*Ae. (Adm.)* undet.	1	1	0	0	0	0	0	**2**
*Ae. (Stg.) *undet.	0	0	1	5	10	0	0	**16**
*Ae. aegypti* ^*^	41	27	1	35	17	9	5	**135**
*Ae. albocephalus*	9	0	0	0	0	9	0	**18**
*Ae. argenteopunctatus*	0	0	3	0	0	0	0	**3**
*Ae. cumminsii*	0	0	0	0	0	4	0	**4**
*Ae. microstictus?*	3	8	0	2	0	0	0	**13**
*Ae. simpsoni*	0	0	0	0	0	1	0	**1**
*Ae. strelitziae*	0	22	0	0	6	1	0	**29**
*Ae. subdentatus*	0	5	0	0	0	14	0	**19**
*Ae. vexans*	0	0	0	0	0	10	0	**10**
*An. tenebrosus*	0	0	0	2	0	0	0	**2**
*An.* undet.	0	0	0	1	0	0	0	**1**
*Cx. (Cux.) *undet.	0	0	1	4	0	4	0	**9**
*Cx. antennatus*	0	0	0	0	0	3	0	**3**
*Cx. chorleyi* ^*^	4	0	0	0	0	0	0	**4**
*Cx. cinerellus*	2	0	0	0	0	0	0	**2**
*Cx. neavei* ^*^	96	5	0	240	5	49	1	**396**
*Cx. nebulosus* ^*^	0	7	5	2	2	8	1	**25**
*Cx. pipiens s.l.* ^*^	237	6	1	147	93	238	0	**722**
*Cx. poicilipes* ^*^	20	1	0	65	0	0	0	**86**
*Cx. rubinotus*	0	0	0	0	0	2	0	**2**
*Cx. simpsoni*	0	1	0	38	3	7	0	**49**
*Cx. sitiens*	0	0	0	0	0	1	0	**1**
*Cx. trifilatus?*	0	0	0	0	0	1	0	**1**
*Cx. tritaeniorhynchus*	0	0	0	0	0	2	0	**2**
*Cx. vansomereni* ^*^	0	1	0	0	0	0	0	**1**
*Er. quinquevittatus* ^*^	13	17	2	2	2	14	0	**50**
*Lt. tigripes*	0	3	0	0	0	0	0	**3**
*Ma. africana*	9	0	0	2	1	0	0	**12**
*Ma. uniformis*	3	0	0	0	0	0	0	**3**
Undet. culicines	3	0	0	0	0	0	0	**3**
*Wy. mitchellii* ^*^	204	0	0	0	0	0	0	**204**
Totals	**645**	**104**	**14**	**545**	**139**	**377**	**7**	**1831**

Sites: DBG: Durban Botanical Gardens; MWNR: Marrianwood Nature Reserve; AC: Albinia Conservancy; JG: Japanese Gardens; Ver: Verulam; BBNR: Burman Bush Nature Reserve; CROW: Centre for Rehabilitation of Wildlife. Genera: Ae.: *Aedes*; An.: *Anopheles*; Cx.: *Culex*; Er.: *Eretmapodites*; Ma.: *Mansonia*; Lt.: *Lutzia*; Wy. *Wyeomyia*. Subgenera: Adm.: *Aedimorphus*; Stg.: *Stegomyia*; Cux.: *Culex*. Undet. indicates undetermined identification on specified taxonomic level. s.l.: sensu lato.

? indicates a level of uncertainty in species determination.

* indicates the detection of an arbovirus.

**Table 4:** Details for each mosquito pool from which a positive amplicon was detected.

Virus^a^	Pool number	VBD^b^ number	Mosquito species	No of mosquitoes per pool	Date collected	Site^c^
SINV	14-21	230/22/08	*Cx. nebulosus*	1	June 2021	BBNR
WITV	AC09	61/21/06	*Er. quinquevittatus*	2	Mar 2021	AC
WITV	BB19	61/21/25	*Cx. neavei*	4	Nov 2020	BBNR
WITV	VER20	61/21/186	*Ae. aegypti*	7	May 2021	Ver
GERV	BB02	61/21/08	*Cx. pipiens s.l.*	4	Oct 2020	BBNR
GERV	BG14	61/21/72	*Cx. poicilipes*	8	Dec 2020	DBG
GERV	JG41	61/21/132	*Ae. aegypti*	1	Mar 2021	JG
BUNV	BG21	61/21/79	*Cx. neavei*	13	Feb 2021	DBG
BUNV	JG03	61/21/100	*Cx. poicilipes*	8	Oct-Nov 2020	JG
BUNV	JG22	61/21/117	*Cx. neavei*	96	Dec 2020	JG
BUNV	VER07	61/21/181	*Cx. pipiens s.l.*	60	Dec 2020	Ver
BUNV	22-21	230/22/15	*Ae. aegypti*	1	May 2021	DBG
BUNV	31-21	230/22/22	*Cx. chorleyi*	4	June 2021	DBG
BUNV	39-21	230/22/29	*Cx. vansomereni*	1	June 2021	MNR
BUNV	85-21	230/22/69	*Wy. mitchellii*	1	June 2021	DBG

^a^ SINV: Sindbis virus; WITV: Witwatersrand virus; GERV: Germiston virus; BUNV: Bunyamwera virus.

^b^ VBD: designated laboratory number.

^c^ BBNR: Burman Bush Nature Reserve; AC: Albinia Conservancy; JG: Japanese Gardens; DBG: Durban Botanic Gardens; Ver: Verulam; MNR: Mariannwood Nature Reserve^**d**^.

Positive pools were noted from mosquitoes collected between November and June, a wide range of months including summer and the beginning of winter. However, the climate in this region remains mild throughout the year and mosquito populations remain active.

### Nucleotide sequencing and confirmation of virus species

The nucleotide sequences of each amplicon from RT-PCR positive pools were determined using MinION sequencing. Each virus species was identified from the nucleotide sequence obtained using Basic Local Alignment Search Tool (BLAST) (https://blast.ncbi.nlm.nih.gov/Blast.cgi) and the results listed in Table 4.

### Similarity analysis

**Fig 3:**
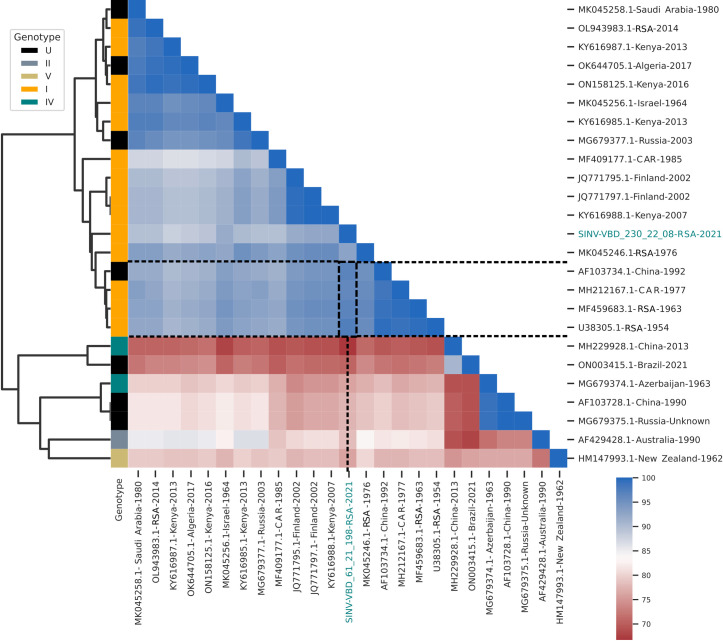
Clustermap for Sindbis virus (SINV) isolates based on a 189 nucleotide region of the Nsp4 encoding gene. Isolates for which genotypes were confirmed in published studies are indicated in the key. SINV VBD 230/22/08 clusters next to genotype 1 isolates highlighted in blue.

SINV RNA was amplified from sample designated VBD 230/22/08 which was comprised of only one mosquito, *Cx nebulosus*, collected from BBNR. A similarity analysis was performed for the SINV isolate based on the partial sequence data obtained from the nsP4 gene and compared with data retrieved from GenBank for isolates from geographically similar and distinct (S3 Table). Fig 3 shows the clustermap obtained for SINV isolates from each genotype I, IV, V and VI. Data for genotypes II and III were not available. A cluster map was used to investigate similarity due to the short length of the amplicon and limited data available. The isolate was identified as clustering with isolates from genotype I, showing the highest similarity with previous South African isolates, Girdwood, SAAR6071 and SAAR86.

**Fig 4:**
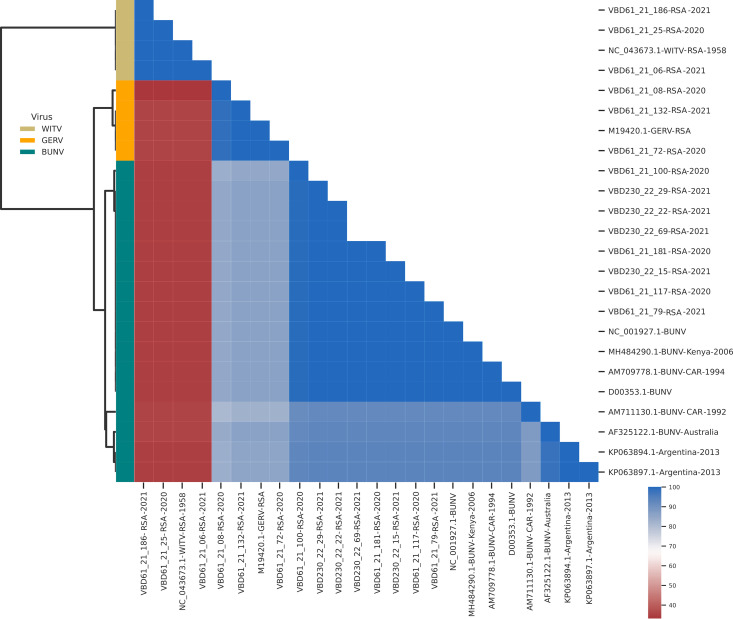
Clustermap for orthobunyaviruses based on 184 nucleotide region of the S segment for various isolates belonging to the genus *Orthobunyavirus*, including Bunyamwera virus (BUNV), Germiston virus (GERV) and Witwatersrand virus (WITV).

Orthobunyavirus RNA was amplified from 14 mosquito pools (Table 4). The species of each isolate was confirmed from the nucleotide sequence data obtained using BLASTn search (Table 4). A similarity analysis was performed for the orthobunyaviruses based on the partial sequence data obtained from the partial S gene and compared with data retrieved from GenBank for isolates from geographically similar and distinct (S4 Table). Fig 4 shows the clustermap obtained for the orthobunyavirus isolates.

## Discussion

Vector surveillance studies play an important role in informing on spatiotemporal distribution of vectors and vector-borne pathogens, and in-so-doing generate disease/outbreak risk profiles for a region. The outcomes can also contribute towards prediction of outbreaks and can be used to facilitate implementation of effective control strategies, vector management, and monitoring of effectiveness of control measures [25]. Here, we have shown the circulation of several zoonotic arboviruses in a major South African metropole, in the presence of established vectors, and recorded, for the first time, an introduced mosquito species *Wyeomyia mitchellii*, harbouring arbovirus RNA.

### *Wyeomyia mitchellii* as an introduced species

*Wyeomyia* Theobald is a genus of New World mosquitoes comprising 139 species in 17 subgenera. *Wyeomyia mitchellii*, often referred to as the bromeliad mosquito, was originally described from Jamaica, even though southern Florida is also considered part of its native range [26]. The species has since been recorded from the Greater Antilles islands and eastern Mexico and is thus considered a tropical and subtropical species of the New World. It has also been introduced to the Hawaiian Islands in the Central Pacific Ocean as well as Tahiti and Moorea, two islands of the Society Islands, French Polynesia, in the South Pacific [22] as well as the Micronesian Island of Guam in the western Pacific [27]. It has thus not been recorded from the continental Old World and this report is the first for both the genus and species.

The females of *Wy. mitchellii* are active in shaded areas during daytime and will readily feed on mammals, including humans [26]. Even though the species has not been investigated as a competent arbovirus vector, Venezuelan equine encephalitis virus (family *Togaviridae*, genus Alphavirus, VEE) has previously been detected in the species [28] as well as BUNV reported here. The species is likely not of great public health concern due to its ontology and ethology [29]. Slow immature development and low adult fecundity that results in slow population growth coupled with limited dispersal by adults, as well as high oviposition/breeding specificity, are generally not characteristics of invasive mosquitoes of public health concern [29].

This is evident from the localised distribution of the species, which seemingly limited to the Durban Botanical Gardens, even though it was likely already introduced in the 1970’s. The Durban Botanical gardens have a rich bromeliad (*Bromeliaceae*) collection, likely developed by Ernest Thorpe who was Curator of the Gardens from 1950 to 1975. The Botanical Gardens also boast with a large, landscaped bromeliad garden planted/installed in 1972 (pers. comm. Martin Clement — current curator, 2024). The importation of these bromeliads, which are also the preferred breeding sites for *Wy. mitchellii,* were the likely the route of introduction and establishment of a founder population.

### Detection of mosquito-borne arboviruses in eThekwini

SINV was detected in a mosquito collected from *Cx. nebulosus* from Burman Bush Nature Reserve. Based on previous phylogenetic analysis of SINV from geographically distinct regions, six genotypes designated I to VI, have been identified [30]. SINV genotype I (SINV-I) are the only members that have been associated with febrile disease and have been isolated from multiple countries in Europe, Africa and the Middle East. Other genotypes are yet to be associated with any disease in humans with SINV-II and VI circulating in Australia, SINV-III in southeast Asia, SIN-IV in the Middle East and Asia and SINV-V (also referred to as Whataroa virus) in New Zealand [31]. The virus circulates between mosquitoes and birds with humans as accidental dead-end hosts. Bird migration between Africa, either from Central Africa or South Africa, and Europe was likely the route of introduction of this virus into Europe where cases are seen annually, especially in Scandinavian countries. The virus has been detected in SA previously and is known to cause sporadic outbreaks in the country usually after periods of heavy rainfall favouring mosquito breeding [32].

Due to the small size of the amplicon and therefore limited sequence data available for each arbovirus isolate amplified from the mosquitoes, a cluster map was used to investigate similarity with known isolates using sequence data retrieved from GenBank. SINV (VBD 230/22/08) grouped with other isolates from Africa and Europe within genotype I, specifically with South African isolate S.A.AR86 and Girdwood. VBD 230/22/08 was detected from *Cx. nebulosus* mosquitoes collected in June 2021. *Culex univittatus* is considered the primary vector for SINV however other species, *Cx neavei* (recently separated from *Cx univittatus*), *Cx pipiens*, *Cx torrentium*, *Cs. morsitans*, *Cq. fuscopennata* are listed as common vectors [1].

WITV was amplified from each of three mosquito pools, including the following species, *Cx. neavei, Ae. aegypti* and *Er. quinquevittatus*. Little is known about this virus except that it was first isolated from *Cx. rubinotus*, in South Africa. However, the competence of this species and other vectors is yet not known [1]. Since this virus was isolated from three different species-pools from three different localities warrants further investigation including vector competency studies to determine its medical and/or veterinary significance.

BUNV was isolated from multiple different mosquito species including *Cx. poicilipes, Cx. neavei, Cx. pipiens s.l., Cx. chorleyi, Cx. vansomereni*, *Ae. aegypti* and *Wy. mitchellii*. BUNV has a wide distribution in Africa and was first isolated from *Aedes* spp. in the Semliki Forest, Uganda [1]. Although the virus has been associated with both culicine and anopheline mosquitoes, flood water *Aedes* spp. are considered the primary vectors [1,33]. Related viruses, Batai and Ngari, have similarly been associated with a wide range of species of mosquitoes but it is likely that not all these mosquitoes are competent vectors involved in maintenance or transmission, and instead may have incidentally fed on infected hosts [33,34]. BUNV is considered endemic in most African countries and has been detected in South America, in Argentina [34]. BUNV has previously been associated with mild disease in humans, and to date, there are no reports to date of severe and/or fatal human disease, although the virus has been isolated from horses with neurological disease and from and aborted equine fetus [34,35]. It is worth noting that this virus is considered parental to Ngari virus, a natural reassortment between BUNV and Batai virus which causes severe haemorrhagic fever, including fatalities, in Kenya [36].

Estimations of local viral activity in this study were not considered accurate due to the opportunistic sampling approach where only a subset of the mosquitoes collected were screened for evidence of infection with arboviruses. Different traps used at different locations introduced biases in the collection of different species which cannot be accounted for in prevalence estimations. The scope of the study was to determine if arboviruses, previously detected in the country from historical surveillance studies, were currently circulating. Further studies will be designed to focus on determining more accurate viral activity patterns and prevalence.

The similarity of BUNV, GERV and WITV were compared with other orthobunyaviruses. The sequence data for the BUNV isolates obtained in the study had high similarity with isolates from geographically distinct regions. however, this is a highly conserved region within BUNV genome and further attempts to obtain sequence data from a more diverse region will be required to investigate diversity.

The sequence data for the GERV and WITV isolates obtained in the study had high similarity with other isolates from southern Africa. Neither GERV, nor WITV, has received much scientific attention. GERV, initially isolated from culicine mosquitoes in SA, has been detected in Botswana, Namibia and Zimbabwe [1]. The primary vectors of GERV are thought to be *Cx. rubinotus* and possibly *Cx. theileri.* Two laboratory acquired infections have previously been described and serological evidence of human infections has been reported from retrospective surveillance studies [1,10]. Isolations of WITV from mosquitoes have been reported from Mozambique, South Africa and Zimbabwe and the primary vector thought be *Cx. rubinotus* [1,37]. No human cases of disease caused by WITV have been reported. Except for the mosquito species from which the isolates were obtained, there is limited knowledge regarding competent vector species for GERV and WITV.

The full clinical spectrum of BUNV, WITV, and GERV is unknown and warrants further investigation. Although BUNV and GERV have previously been associated with mild disease, the true burden of disease and disease profiles may be underestimated. The detection of these viruses in mosquito pools confirms current circulation of each viral species with high similarity to previously detected isolates in southern Africa. Human cases have not recently been reported however in the absence of awareness and diagnostic capacity it is not possible to exclude occurrence of human infection. Vector surveillance studies contribute towards identifying circulating pathogens in vector hosts but need to be complemented with retrospective and prospective screening of human populations to profile associated diseases and fully understand the burden of disease, if any, that is caused by lesser-known arboviruses. The detection of these viruses in multiple mosquito vectors does not imply that all these species are competent vectors. Detection could merely be from recent blood meals or spill over into other mosquito populations that are circulating and feeding in the same area. Vector competency studies would be required to determine the role of other mosquitoes in the transmission of these viruses and their role in maintenance. The mosquitoes were collected during the rainfall season when mosquito populations are abundant in KZN, which has a mild climate, subtropical latitude and warmer humid weather favouring mosquito breeding. Despite the study limitations regarding the small number of mosquitoes available for screening and amplification of partial gene fragments that did not permit robust phylogenetic analysis, we were able to confirm the presence of lesser-known arboviruses in the region that have not recently been reported. Detection of SINV was not unexpected as the virus is known to occur in SA and causes outbreaks of human disease.

Overall, the detection of multiple viruses, and a previously undocumented mosquito species, emphasizes the need for surveillance studies, more accessible diagnostics and raised awareness of arboviral infections.

## Supporting information

S1 Table *Alphavirus* and *Orthobunyavirus* sequence data used for primer design. GenBank accession numbers for the *Alphavirus* and *Orthobunyavirus* sequence data that were used for primer design.
(DOCX)

S2 Table Partial cytochrome c oxidase 1 gene obtained for mosquito identification. BLASTn analysis, identity 683/687 (99%) *Wyeomyia mitchelli* M4 mitochondrial COX1 gene (3 gaps).
(DOCX)

S3 Table GenBank accession numbers for *Alphavirus* sequences. GenBank accession numbers for *Alphavirus* sequence data that were used for the identity matrix visualised as a clustermap (Fig 3).
(DOCX)

S4 Table GenBank accession numbers for *Orthobunyavirus* sequences. GenBank accession numbers *Orthobunyavirus* sequence data that were used for the identity matrix visualised as a clustermap (Fig 4).
(DOCX)

S5 Table Location of collection sites. GPS coordinates for each collection site in eThekwini, KZN.
(DOCX)
