# Supplementary material for: Identification of arboviruses in mosquito populations in KwaZulu-Natal, South Africa and the first record of Wyeomyia mitchellii in the Old World
Source: PLoS Negl Trop Dis. 2025 Aug 12;19(8):e0013093. doi: 10.1371/journal.pntd.0013093 (PMC12342292; doi:10.1371/journal.pntd.0013093)
Supplement: S1 Table — GenBank accession numbers for the Alphavirus and Orthobunyavirus sequence data that were used for primer design. (DOCX) [file pntd.0013093.s001.docx]

**Supplementary data**

**S1 Table *Alphavirus* and *Orthobunyavirus* sequence data used for primer design**

| **Genus** | **Virus** | **GenBank Accession numbers** |
| --- | --- | --- |
| *Alphavirus* | Sindbis virus | MK045247.1; MK045246.1; U38304.1; MK045250.1; U38305.1 |
|  | Ndumi virus | JX644171.1; AF339487.1; JN989958.1; JX644169.1; JX644167.1; JX644170.1; JX644168.1; JX644166.1 |
|  | Middelburg virus | AF339486.1; KM115530.1; KM115531.1; KF680222.1; EF536323.1 |
|  | Chikungunya virus | KF283988.1; MK280688.1; Z48163.2; Y14761.1 |
|  | Semliki forest virus | HQ456255.1; HQ456254.1; HM045793.1; HM045822.1; HM045823.1; HM045812.1; HM045784.1; HM045809.1; HM045805.1; HM045795.1; HM045821.1; HM045792.1; HM045811.1 |
|  | O’nyong’nyong virus | M20303.1; KX771232.1; AF079456.1; MF409176.1; AF079457.1 |
| *Orthobunyavirus* | Bunyamwera virus | AF325122.1; AM709778.1; AM711130.1; KP063894.1; KP063897.1; KP063900.1; MH484290.1; NC001927.1 |
|  | Germiston virus | M19420.1 |
|  | Shuni virus | MF361855.1; MF361852.1; MF361849.1; MF361846.1; KT946779.1; KP900883.1; KP900872.1; KP900878.1; KP900867.1; KU937313.1; HE800143.1 |
|  | Witwatersrand virus | NC_043673.1 |
