# Supplementary material for: Identification of arboviruses in mosquito populations in KwaZulu-Natal, South Africa and the first record of Wyeomyia mitchellii in the Old World
Source: PLoS Negl Trop Dis. 2025 Aug 12;19(8):e0013093. doi: 10.1371/journal.pntd.0013093 (PMC12342292; doi:10.1371/journal.pntd.0013093)
Supplement: S2 Table — BLASTn analysis, identity 683/687 (99%) Wyeomyia mitchelli M4 mitochondrial COX1 gene (3 gaps). (DOCX) [file pntd.0013093.s002.docx]

**S2 Table: Partial cytochrome c oxidase 1 gene obtained for mosquito identification. BLASTn analysis, identity 683/687 (99%) *Wyeomyia mitchelli* M4 mitochondrial COX1 gene (3 gaps)**

GGTCAACAAATCATAAAGATATTGGAACTTTATATTTTATTTTTGGTGCTTGATCAGGAATAGTAGGAACTTCTTTAAGAATTTTAATTCGAACTGAATTAAGTCATCCAGGGGCATTTATTGGAAATGACCAAATTTATAATGTTATTGTTACAGCTCATGCATTTATTATAATTTTTTTTATAGTAATACCTATTATAATTGGAGGATCGGAAATTGACTAGTTCCATTAATATTAGGAGCACCTGATATAGCCTTTCCACGAATAAATAATATAAGTTTTTGACTTCTTCCTCCTTCTTTAACATTATTATTAGCCGGAAGAATAGTAGAAAATGGAGCTGGGACTGGATGAACTGTTTATCCTCCTCTATCTTCCAATTTAGCTCATACAGGAGCTTCAGTAGATCTTTCAATTTTTTCTCTTCATTTAGCAGGAATTTCCTCTATTTTTAGGAGCAGTAAATTTTATTACTACTGTAATTAATATACGATCTACTGGAATTACTTTAGATCGAATACCTTTATTTGTTTGATCTGTTGTAATTACAGCTATTTTATTACTTCTTTCCCTTCCTGTTTTAGCTGGAGCTATTACCATATTATTAACAGATCGAAATTTAAATACTTCATTCTTTGATCCAATTGGAGGAGAGACCCTATTCTTTATCAACATTTATTTTGATTTTTTGGTCACCCTGAAGTTTA
