# Supplementary material for: Identification of arboviruses in mosquito populations in KwaZulu-Natal, South Africa and the first record of Wyeomyia mitchellii in the Old World
Source: PLoS Negl Trop Dis. 2025 Aug 12;19(8):e0013093. doi: 10.1371/journal.pntd.0013093 (PMC12342292; doi:10.1371/journal.pntd.0013093)
Supplement: S3 Table — GenBank accession numbers for Alphavirus sequence data that were used for the identity matrix visualised as a clustermap (Fig 3). (DOCX) [file pntd.0013093.s003.docx]

**S3 Table. GenBank Accession numbers for alphavirus sequences**

| **Accession number** | **Isolate (I) or Strain (St)** | **Genotype** | **Country of isolation** | **Collection date** |
| --- | --- | --- | --- | --- |
| PV991052.1 | I: VBD 230/22/08 | Unknown | South Africa | 2021 |
| AF103728.1 | St: XJ-160 | Unknown | China | 1990 |
| AF103734.1 | St: YN87448 | Unknown | China | 1992 |
| AF429428.1 | I: SW6562 | II | Australia | 1990 |
| HM147993.1 | St: Whataroa | V | New Zealand | 1962 |
| JQ771795.1 | I: Ilomantsi-2002B | I | Finland | 2002 |
| JQ771797.1 | I: Johannes-2002 | I | Finland | 2002 |
| KY616985.1 | I: BONI_584_KENYA_2013 | I | Kenya | 2013 |
| KY616987.1 | I: BONI_566_KENYA_2013 | I | Kenya | 2013 |
| KY616988.1 | I: NVS_305_KENYA_2007 | I | Kenya | 2007 |
| MF409177.1 | I: ArB489 | I | Central African Republic | 1985 |
| MF459683.1 | St: Girdwood_S.A. | I | South Africa | 1963 |
| MG679374.1 | St: Kyzylagach_LEIV-65A | IV | Azerbaijan | 1963 |
| MG679375.1 | St: Stavropol | Unknown | Russia | Unknown |
| MG679377.1 | St: LEIV-Ast03-1-839 | Unknown | Russia | 2003 |
| MH212167.1 | St: ArB7761 | I | Central African Republic | 1977 |
| MH229928.1 | St: YN_222 | IV | China | 2013 |
| MK045246.1 | I: SAAR_18141/Cx_univit- tatus/South_Africa/1976 | I | South Africa | 1976 |
| MK045256.1 | I: 1038/Streptopelia_turtur/ Israel/1964 | I | Israel | 1964 |
| MK045258.1 | I: SA80_394/Cx_univittatus/ Saudi/1980 | Unknown | Saudi Arabia | 1980 |
| OK644705.1 | I: P29_Algeria | Unknown | Algeria | 2017 |
| OL943983.1 | I: GAU14MP070/SA | I | South Africa | 2014 |
| ON003415.1 | I: LMP_A | Unknown | Brazil | 2021 |
| ON158125.1 | I: KCH_SS4_486 | I | Kenya | 2016 |
| U38305.1 | I: S.A.AR86 | I | South Africa | 1954 |
