# Supplementary material for: Identification of arboviruses in mosquito populations in KwaZulu-Natal, South Africa and the first record of Wyeomyia mitchellii in the Old World
Source: PLoS Negl Trop Dis. 2025 Aug 12;19(8):e0013093. doi: 10.1371/journal.pntd.0013093 (PMC12342292; doi:10.1371/journal.pntd.0013093)
Supplement: S4 Table — GenBank accession numbers Orthobunyavirus sequence data that were used for the identity matrix visualised as a clustermap (Fig 4). (DOCX) [file pntd.0013093.s004.docx]

**S4 Table. GenBank Accession numbers for orthobunyavirus sequences**

| **Accession number** | **Virus** | **Isolate (I) or Strain (St)** | **Country of isolation** | **Collection date** |
| --- | --- | --- | --- | --- |
| PV891240.1 | BUNV | I: VBD 61/21/100 | South Africa | 2020 |
| PV891241.1 | BUNV | I: VBD 61/21/117 | South Africa | 2020 |
| PV891243.1 | BUNV | I: VBD 61/21/181 | South Africa | 2020 |
| PV891242.1 | BUNV | I: VBD 230/22/15 | South Africa | 2021 |
| PV891245.1 | BUNV | I: VBD 230/22/22 | South Africa | 2021 |
| PV891246.1 | BUNV | I: VBD 230/22/29 | South Africa | 2021 |
| PV891244.1 | BUNV | I: VBD 230/22/69 | South Africa | 2021 |
| PV891239.1 | BUNV | I: VBD 61/21/79 | South Africa | 2021 |
| AF325122.1 | BUNV | Unknown | Australia | Unknown |
| AM709778.1 | BUNV | St: ArB29051 | Central African Republic | 1994 |
| AM711130.1 | BUNV | St: ArB28215 | Central African Republic | 1992 |
| D00353.1 | BUNV | Unknown | Unknown | Unknown |
| KP063894.1 | BUNV | St: SFCrEq231 | Argentina | 2013 |
| KP063897.1 | BUNV | St: SFBzEq232 | Argentina | 2013 |
| MH484290.1 | BUNV | St: 46A-122 | Kenya | 2006 |
| NC_001927.1 | BUNV | Unknown | Unknown | Unknown |
| PV991046.1 | GERV | I: VBD 61/21/08 | South Africa | 2020 |
| PV991047.1 | GERV | I: VBD 61/21/72 | South Africa | 2020 |
| PV991048.1 | GERV | I: VBD 61/21/132 | South Africa | 2021 |
| M19420.1 | GERV | Unknown | South Africa | Unknown |
| PV991050.1 | WITV | I: VBD 61/21/25 | South Africa | 2020 |
| PV991049.1 | WITV | I: VBD 61/21/06 | South Africa | 2021 |
| PV991051.1 | WITV | I: VBD 61/21/186 | South Africa | 2021 |
| NC_043673.1 | WITV | St: SAAr 1062 | South Africa | 1958 |
