# Supplementary material for: Identification of arboviruses in mosquito populations in KwaZulu-Natal, South Africa and the first record of Wyeomyia mitchellii in the Old World
Source: PLoS Negl Trop Dis. 2025 Aug 12;19(8):e0013093. doi: 10.1371/journal.pntd.0013093 (PMC12342292; doi:10.1371/journal.pntd.0013093)
Supplement: S5 Table — GPS coordinates for each collection site in eThekwini, KZN. (DOCX) [file pntd.0013093.s005.docx]

**S5 Table: Location of collection sites**

| **Collection Site** | **Latitude** | **Longitude** |
| --- | --- | --- |
| Albinia Conservancy | 29.772211°S | 30.779422°E |
| Burman Bush Nature Reserve | 29.817701°S | 31.017238°E |
| Centre for the Rehabilitation of Wildlife (CROW) | 29.91954°S | 30.93628°E |
| Durban Botanic Gardens | 29.844874°S | 31.006623°E |
| Japanese Gardens | 29.795517°S | 31.039401°E |
| Marrianwood Nature Reserve | 29.834694°S | 30.839106°E |
| Verulam | 29.63333°S | 31.05°E |
